# Marker-Less 3d Object Recognition and 6d Pose Estimation for Homogeneous Textureless Objects: An RGB-D Approach

**DOI:** 10.3390/s20185098

**Published:** 2020-09-07

**Authors:** Nasim Hajari, Gabriel Lugo Bustillo, Harsh Sharma, Irene Cheng

**Affiliations:** Multimedia Research Centre, Department of Computing Science, University of Alberta, Edmonton, AB T6G 2R3, Canada; lugobust@ualberta.ca (G.L.B.); hsharma@ualberta.ca (H.S.); locheng@ualberta.ca (I.R.)

**Keywords:** 6d pose estimation, 3d object recognition, textureless objects, homogeneous objects

## Abstract

The task of recognising an object and estimating its 6d pose in a scene has received considerable attention in recent years. The accessibility and low-cost of consumer RGB-D cameras, make object recognition and pose estimation feasible even for small industrial businesses. An example is the industrial assembly line, where a robotic arm should pick a small, textureless and mostly homogeneous object and place it in a designated location. Despite all the recent advancements of object recognition and pose estimation techniques in natural scenes, the problem remains challenging for industrial parts. In this paper, we present a framework to simultaneously recognise the object’s class and estimate its 6d pose from RGB-D data. The proposed model adapts a global approach, where an object and the Region of Interest (ROI) are first recognised from RGB images. The object’s pose is then estimated from the corresponding depth information. We train various classifiers based on extracted Histogram of Oriented Gradient (HOG) features to detect and recognize the objects. We then perform template matching on the point cloud based on surface normal and Fast Point Feature Histograms (FPFH) to estimate the pose of the object. Experimental results show that our system is quite efficient, accurate and robust to illumination and background changes, even for the challenging objects of Tless dataset.

## 1. Introduction

Recognising an object and estimating its 6d pose simultaneously has received considerable attention in recent years. Object recognition is the process of classifying the instances of real-world objects in a scene. The process typically extracts distinct features from the Region of Interest (ROI) and feeds the extracted features to a previously trained model to recognise the object’s category. Pose estimation is the process of finding the pose of a known 3d object in the scene with respect to the camera. Usually, the pose of a rigid body object is described by 6 Degree of Freedom (DOF) transformation matrix, which consists of three translation and three rotation parameters. Hence it is called 6d pose estimation. Applications such as autonomous driving, augmented reality and robotics vision have a very strong need for accurate and efficient recognition and pose estimation approaches. As these methods are mainly based on the shape and model characteristics of the objects, they are called model based approaches [1]. They can be divided into global and local techniques based on their descriptors. Global approaches describe the whole object using a global descriptor. These techniques need a segmentation step and therefore are not very suitable for occluded scenes. On the other hand, the local approaches describe the objects by using local descriptors around specific points and are more sensitive to the sensor noise and less accurate for symmetric textureless objects. Guo et al. [2] discussed 3d local feature descriptors and evaluated a number of techniques, including Spin Images [3], Fast Point Feature Histograms (FPFH) [4] and Signature of Histograms [5].

The object detection problem has been well addressed for textured, distinctive objects, where the objects can be represented by a sparse set of features such as Scale Invariant Feature Transform (SIFT) [6], Speeded Up Robust Features (SURF) [7] or Oriented FAST and Rotated BRIEF (ORB) [8]. There is an increasing demand to accurately detect simple, symmetrical and textureless objects. Manufacturing and production industrial parts are such examples where a robotic system needs to perform some commonplace tasks such as pick-and-place or object inspection. Understanding the 6d pose of an object will facilitate the end effector in picking up the object and performing the required tasks. Textureless objects can best be described by their global shape features, such as edges and depth cues [9,10,11,12]. The detection methods based on photometric local patches such as [13,14] will fail for these objects. Another approach for shape retrieval is proposed in [15], where the shape is partitioned into multiple level of curve segments. The highest level of the descriptor captures the global properties of the shape, while the detailed information of the shape is captured at the finer levels. To improve the time performance of shape retrieval approaches, researchers [16] suggested to move the heavy computation processes to the offline stages. With the affordable price and accessibility of the RGB-D sensors, researchers proposed different object detection and pose estimation methods using both optical and depth information [10,17,18,19,20]. Although these methods usually outperform the approaches based on optical information only, depth sensors have a limited capturing angle and are more sensitive to illumination conditions. Therefore, other researchers estimated the 6d pose from 2d images [21,22,23,24]. In this paper we present a framework to simultaneously recognise the object’s class and estimate its 6d pose from RGB-D data. The proposed model is a global approach, where the object or the ROI is first recognised from the RGB image. The pose will be estimated from the corresponding depth information. We train a Stochastic Gradient Descent (SGD) classifier based on extracted Histogram of Oriented Gradient (HOG) features to detect and recognise the objects. We then perform template matching on the point cloud based on surface normal and Fast Point Feature Histograms (FPFH) to estimate the pose of the object. Experimental results show that our system is accurate, efficient and robust to illumination and background changes, even for the challenging objects of Tless [25] dataset. The remainder of this paper is organised as follows: related work is described in Section 2. The proposed method is discussed in Section 3. The details of our experimental setup are presented in Section 4. Section 5 explains our evaluation metrics. We show our experimental results and compare our method with other techniques in Section 6. Finally, Section 7 concludes the paper.

## 2. Related Work

Traditional approach for 3d object recognition is through template matching. Although time performance of template matching is poor and not suitable for real-time applications, studies [9] have shown that the need for training samples is less and it can recognise new objects by comparing with an existing database. The methods proposed by [9,10] are based on efficient template matching. They used the optical images to detect objects. Furthermore, Hinterstoisser et al. [9] showed that occlusion can become less problematic by using depth information. Their feature set contains surface normal and orientations of the contour gradient. These techniques work best for heterogeneous textureless objects. However, these methods are not quite effective on industrial parts, which are usually symmetric and have simple, homogeneous shapes. Region based approaches are used extensively for object recognition and pose estimation on 2d images. Hexner and Hagege [26] used active contour to segment the images into background and foreground. They estimated the object pose by using multiple local appearance models. These models can capture spatial variations and therefore work best for heterogeneous objects.Brachmann et al. [24] proposed a framework to estimate the 6d pose of an object from a single RGB image. They reduced the uncertainty in object coordinates and object prediction iteratively. In order to deal with missing depth information, they marginalised the object coordinate distribution over depth. Another region based approach was proposed in [27], where the authors estimated the pose of an object using the local colour histogram of a single RGB image. In case the objects do not have colour or textural information, this approach fails to produce reliable results. More recently, people used different Artificial Neural Networks (ANN) for 3d object recognition and 6d pose estimation [18,19,28,29]. Gupta et al. [18] used both colour images and depth features to train a Convolutional Neural Network (CNN) model. Depth features gave information about the horizontal disparity, height above the ground and angle with gravity for each pixel. Data augmentation is an essential part for learning based models to create a generally richer training set. For instance, authors of [20] augmented the data to create a rich training set for autonomous navigation. Capturing enough datasets or augmenting the data for 6d pose estimation applications can be extensively time consuming. Some researchers [13,30] proposed a robust and scalable object detection technique which combines CNN with region-based approach to localise and detect the objects. They call this network R-CNN. Later, other researchers modified and used this network to recognise and detect the objects for autonomous driving and object localisation [14,31,32,33,34,35,36,37,38]. However, almost all of these networks rely heavily on the textural information of the objects. The network proposed by [38] can handle irregular shape textureless objects. However, this is not a robust technique for industrial parts where different parts can be very similar in shapes. Mahendran et al. [36] proposed that a CNN regression framework is more promising than a CNN classifier framework for 6d pose estimation. The reason is that pose characterisation is a continues space and therefore pose estimation problem is a “continues” computation problem in nature. The authors in [22] predict the pose of objects using RGB images only. They first segmented the 2d images to localise the object of interest and then used a CNN model to predict the pose of the object. However, since they did not use any depth information or depth cues, they could not predict the full 6d pose of the object. Some researchers modified the 2d object detector networks to recognise 3d models and estimate the 6d pose. The Single Shot Descriptor (SSD) object detection network was modified by [23] to detect 3d models and estimate their 6d poses using a single RGB image. Simon et al. [39] modified YOLO v2 network, which is a real-time 2d object detector, to add a regression strategy to estimate multiclass bounding cubes. This network works on 3d point clouds. However, point clouds are usually noisy and capturing a clean one requires more expensive computation power. DeepIM introduced in [40] to estimate the 6d pose of the objects using RGB images. The idea is to match the rendered image of a 3d model with the input images. It starts from an initial pose and in an iterative manner changes the pose of the rendered image until a good match is found. Sundermeyer et al. [12] proposed object detection and 6d pose estimation from RGB data based on an augmented autoencoder. They defined an implicit representation of object orientation in a latent space. Some people used Hough forests to estimate the object’s pose and predict the next best view [41]. They used a sparse autoencoder to learn features from depth invariant patches automatically. Wohlhart and Lepetit [42] proposed an object detector and 3d pose estimator on poorly textured but heterogeneous objects. They trained a CNN model based on similarity and dissimilarity constraints. Different datasets for 3d object recognition and pose estimation have been introduced in recent years. A public dataset for estimating the 6d pose of textureless, rigid industrial parts, called TLess, was introduced by [25]. This dataset has 30 objects where they bear similarities in shapes or sizes. Some of the objects are parts of other objects in the dataset. The authors showed that objects in TLess are very challenging and even the state-of-the-art techniques fail in different scenarios. In this paper, we focus on this dataset as it resembles our application requirements. Another public dataset of 15 different textureless objects was created by [17]. Although these objects are textureless, they are not industrial related parts and have very distinct geometrical shape features. Due to the distinct geometrical features, this dataset is less challenging compared to [25]. Hodan et al. [43] proposed a benchmark for 6d pose estimation. This benchmark grouped eight different datasets including TLess and presented them in the same format. They also proposed an evaluation method based on a pose-error function. This study showed that methods based on point-pair features and template matching perform better compared to learning based or 3d local feature approaches. Therefore, we adapt a similar approach to point-pair feature for 6d pose estimation.

## 3. Proposed Method

**Figure 1:**
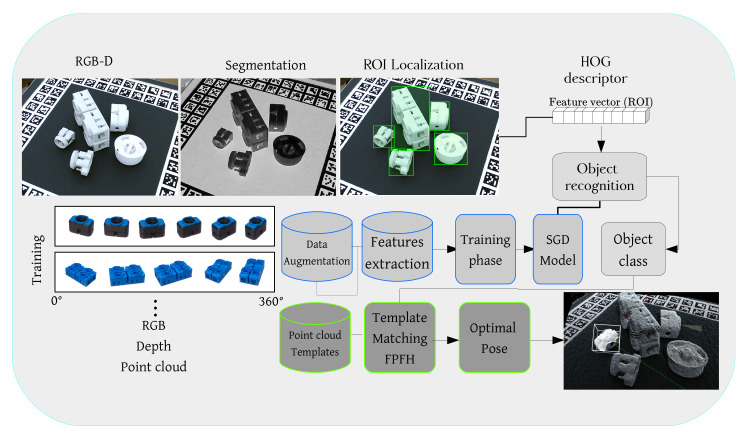
Our object detection and pose estimation pipeline. After object segmentation and localisation (RGB image), each Region of Interest (ROI) is cropped and normalised. HOG features are extracted and forwarded into the SGD object recognition model. After object recognition, we generate the point cloud of the object using both depth and RGB image. We adapt a point cloud matching strategy using the Fast Point Feature Histograms (FPFH) descriptor to find the target object’s optimal pose in our database.

It has been shown that point cloud analysis and feature extraction can be a costly operation and very time consuming, especially if the point cloud is dense [4,44,45]. Researchers have used data reduction techniques to make this process more tractable and close to real-time [46,47]. Others have used different Neural Networks such as Voxnet [48] and So-net [49] for point cloud segmentation and 3d object recognition. These networks are mainly used for a high range environment such as outdoor scenes or living spaces where objects are rich in textural information and have distinctive local and geometrical features. In this paper, we propose a framework for object localisation, recognition and 6d pose estimation using a publicly available and affordable RGB-D camera. To make the system more time efficient and practical, we perform object localisation and recognition on optical data and 6d pose estimation on the point cloud. Figure 1 shows the details and an example of our proposed framework.

**Figure 2:**
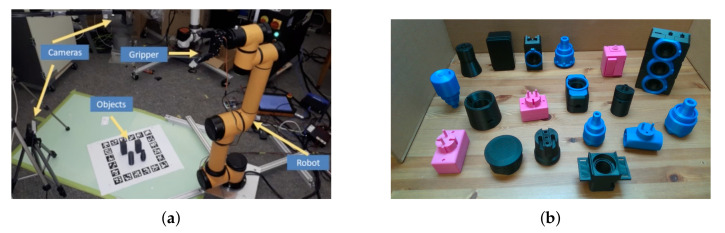
(**a**) Our experimental setup. The images from the top view camera provide an initial estimate of the ROI and the images from the perspective view camera provide the final object detection and recognition. (**b**) Some of the textureless objects we used. All of the objects are 3d printed from [25].

We focus on a pick and place task for homogeneous, similar and textureless industrial parts, which are the most challenging cases [12,27]. Figure 2 shows the experimental setup and some of the objects we used. All the objects in this research are 3d prints of 30 industrial CAD models as introduced in the TLess dataset [25].

### 3.1. Optical Object Localisation

The first step for any object recognition system is to detect and localise the object or ROI. We use two cameras to handle the occlusion problem, one at the top and one at the side of the scene, as Figure 2 shows. The top view camera gives an initial estimate of the number of objects and their locations in the scene. We then use the perspective view camera for the final object detection and recognition. Details on cameras’ setup are discussed in Section 4. We modify the watershed transformation technique proposed by [50] and use it to segment the image to detect the ROI. The algorithm groups the pixels in an image based on their intensity similarity. It floods the image from different sources or markers and segments are determined when different sources meet. Watershed transformation produces closed object contours at low computational cost compared to other more sophisticated vision-based or learning-based segmentation techniques. However, this approach usually oversegments the image because of noise and diverse intensity distribution of the data. Therefore, a good selection of an initial source or marker is crucial. The marker regions are pixels that we can label unambiguously as either object or background. Markers are found at the maximum extreme parts of a histogram. We also flood the gradient image to reduce the effect of different color intensities in the input scene. We use two 3 × 3 Sobel filters to find the gradient amplitudes of the optical image in the horizontal and vertical directions. Even if the markers in the background are not well distributed, the barriers in the elevation map are high enough for these markers to flood the entire background. This technique works best if the objects and background histograms are distinctive. For example, dark color objects on a light background, or vice versa. Otherwise, choosing good markers for background and foreground can be challenging. In this case, background subtraction can provide an initial guess and then the watershed segmentation technique can define the ROI.

**Figure 3:**
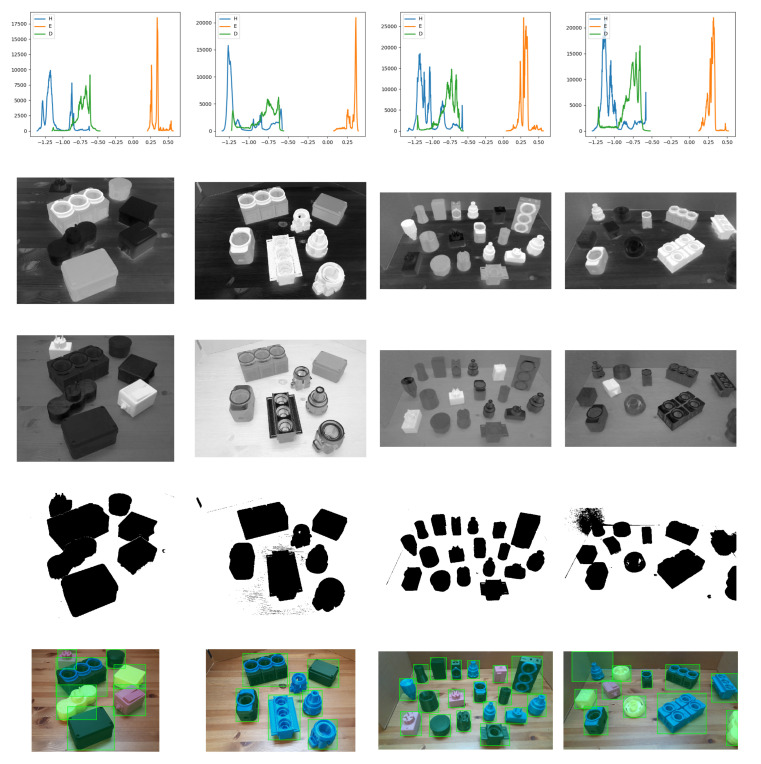
**Top row**: histogram of pixels in Haematoxylin-Eosin-DAB (HED) color space for 4 different scenes; **Second row**: Haematoxylin component of the input RGB image; **Middle row**: Eosin component of the input RGB image; **Fourth row**: binary image with all the segmented objects after applying a threshold value in each HED component; **Bottom row**: bounding box of each region of interest.

In our experiments, there are multiple objects made of semitranslucent color PLA (light yellow), shiny color PLA (pink) or dark color PLA (black and blue). Watershed method does not perform well when objects are translucent or shiny. We solve this problem by adding another segmentation process to generalise object localisation. The RGB input image is passed through two different stages of color space separation to extract the multiple objects from the background. First, a color deconvolution approach by [51] called Haematoxylin-Eosin-DAB (HED) is applied to the RGB image. In this approach each pixel is divided into pure DAB, eosin and hematoxylin stained components. This method is commonly used in the immunohistochemistry (IHC) field for image analysis such as immunisations analysis. This method can supplement object segmentation. A standard 3 by 3 matrix $M_{R}$ separates three-channel and two-channel images. The row vectors ${\vec{H}}_{R}$ (for Hematoxylin), ${\vec{D}}_{R}$ (for DAB) and ${\vec{E}}_{R}$ (for Eosin) are the basis vectors for three color channels (or two channels and a residual). Our hypothesis is that the result of color deconvolution can segment shiny objects in a specific HED component by using a cut-off point value. For the detection of translucent object, a similar process is performed converting the RGB input image to CIE-LAB color space. *L* represents luminance, and *A* and *B* are defined as chromaticity coordinates. The chromaticity *A* and *B* are color directions: +A is the red axis, −A is the green axis, +B is the yellow axis and −B is the blue axis. The histograms of the components (HED and CIE-LAB) are revised to search for a cut-off point to separate ROI objects and accurately delineate them from the background. Light objects contrast against background in the Eosin component, while dark objects are distinguished in the hematoxylin component. Translucent objects are detected in one channel of the CIE-LAB color space. Background pixel values are found in the highest extreme part of the histogram, while ROI pixels are represented by the inferior extremes. Figure 3 shows the underlying histogram of the scenes, different HED channels and the result of our object localisation approach in different crowded scenes. The details of our proposed object localisation algorithm are explained in the pseudocode below (Algorithm 1).

**Algorithm 1** Object Localisation
1:From top view camera2:$[H_{1},E_{1},D_{1}]\leftarrow$ transfer the image into HED space3:$[BB_{1}]\leftarrow$ segmentation($H_{1}$) ∪ segmentation($E_{1}$) ∪ segmentation($D_{1}$)4:From side view camera5:$[H_{2},E_{2},D_{2}]\leftarrow$ transfer the image into HED space6:$[BB_{2}]\leftarrow$ segmentation($H_{2}$) ∪ segmentation($E_{2}$) ∪ segmentation($D_{2}$)7:
**if** $|BB_{1}|=|BB_{2}|$ **then**
8: Use side view images / / there is no occlusion in the side view9:
**else**
10: **for all** BB in $BB_{1}$ **do**11:  $C\leftarrow corner_{BB}$
12:  $match\leftarrow find\_correspondence(C,BB_{2})$
13: **end for**
14:
**end if**
15:**if**match= empty **then**16: Do nothing / / complete occlusion from the side view17:
**else**
18: Divide $BB_{2}$19:
**end if**



### 3.2. Optical Object Recognition

Object recognition techniques is proved to be successful on most of the everyday use objects and natural scenes, which have reasonable resolutions. CNN based approaches such as YOLO [52] and SSD [53] and their variations can satisfactorily detect and recognise most everyday seen objects such as humans and vehicles. However, industrial parts are usually small, textureless and similar, which makes the recognition problem challenging. As our previous study shows [54], some of the objects in the industrial settings are quite similar, with minor differences like a hole or a dent. To create our recognition model, we first extracted some shape related features from the industrial objects and then trained a classifier to label the objects in the scene. We cannot use texture-based feature descriptors due to the textureless characteristics of the industrial objects. We need to rely on shape-based descriptors to capture the small variations in objects. Histogram of Oriented Gradients (HOG), as first introduced by Dalal and Triggs [55], is a shape based feature descriptor effective in capturing the local silhouette characteristics of objects. The formulation of HOG is explained in Equation (1).

(1) $$\begin{array}{c} \nabla_{p_{c}}=\left[\begin{array}{c} \frac{\partial p_{c}}{\partial x}\\ \frac{\partial p_{c}}{\partial y} \end{array}\right]=\left[\begin{array}{c} \left(h_{x}\times p_{c}\right)\\ \left(h_{y}\times p_{c}\right) \end{array}\right],\\ w=\sqrt{{\frac{\partial p_{c}}{\partial x}}^{2}+{\frac{\partial p_{c}}{\partial y}}^{2}},\\ \phi \left(\nabla_{p_{c}}\right)=\arctan\left(\frac{\frac{\partial p_{c}}{\partial y}}{\frac{\partial p_{c}}{\partial x}}\right). \end{array}$$

where $\nabla_{p_{c}}$ is the partial derivative of pixel *p* inside cell *c* of HOG. Each pixel inside the cell casts a weighted vote, *w*, on the corresponding gradient’s bin. ϕ is the gradient direction of each pixel. $h_{x}$ and $h_{y}$ are derivative filters along the horizontal and vertical directions respectively. Each cell then forms a histogram of oriented gradients $\zeta_{c}$. The feature vector ν of the ROI is the concatenation of these histograms for all the cells. The bigger the number of bins or the smaller the cell size, the more features the descriptor provides. The effect of different cell size and bin size on recognition is shown in Section 6. We use four different classifiers including Stochastic Gradient Descent (SGD mini-batch), Perceptron, Passive I and Passive II, to train our object detection model. Passive-aggressive (PA) family algorithms [56,57] are maximum margin based algorithms, which have been mainly used in online learning. PA classifier was originally developed to solve binary classification problems, but these methods can be used for multiclass problems. SGD has been widely used for training linear classifiers in many applications. This algorithm demonstrates high performance in computation time with no loss in classification on large-scale machine learning problems. SGD minimises an objective function by approximating the gradient for a randomly selected batch from the training data. In each iteration, SGD considers one sample and updates the weight vector using a time-dependent weighting factor until it converges. Perceptron is a linear classifier algorithm similar to SGD. It predicts based on a linear function combining a set of weights with the input feature vector. It has shown high performance for large datasets and can be used in online learning. Perceptron classifiers are faster in training. The algorithm is not regularised or penalised and converges to an optimal solution for linearly separable patterns with no upper bound on the learning rate parameter. For a large amount of data classification, this type of learning is preferable. On the other hand, the mini-batch gradient descent method uses *b* (i.e., the mini-batch size) samples at each update, which is suitable for large dataset in online learning. In our case, a feature vector containing HOG provides the samples. Let us represent the size of mini-batch and the total number of training data with Q and M, respectively. For the mini-batch gradient descent, there are a total of T=M/Q iterations per training cycle or epoch. The weight parameter w, which is obtained through optimisation of the approximated value of the error function *f*, is defined as:

(2) $$E\left[f\left(w\right)\right]=\frac{1}{Q}\sum_{i=(t-1)Q+1}^{tQ}f\left(w;x_{i}\right)$$

where t∈1,…,T is the iteration index and $x_{i}$ is the *i*th training sample. At each iteration the weights are adjusted using the gradient descent update rule:

(3) $$w^{t+1}=w^{t}-\varphi \nabla_{w}E_{t}\left[f\left(w^{t}\right)\right]$$

with φ being the learning rate and, $\nabla_{w}E_{t}$ gradient of the loss function. We will discuss the experimental results in Section 4.

### 3.3. Point Cloud Based Pose Estimation

**Figure 4:**
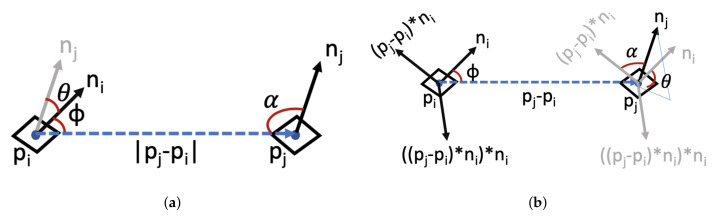
Representation of 4d feature set for Point Pair Features (PPF) and PFH approaches. The features are the distance between two selected surface points ($p_{i}$ and $p_{j}$), and α, Φ and θ angles (**a**) PPF setting, (**b**) PFH setting.

For a robotic arm to interact with an object and pick and place it in its designated location, the 6d pose of the object should be known. The 6d pose is the 3d rotation and 3d translation information with respect to a reference point. Different approaches have been proposed and studied over the past few years. Hodaň et al. [43] proposed a benchmark consisting of eight different datasets to compare various methods based on their performances. They grouped the pose estimation methods into four different classes: learning based, template matching, Point Pair Features (PPF) and 3d local feature methods. In a learning based method such as [24,58,59], each pixel or patch of the ROI in RGB or RGB-D images predicts the 6d pose of the object based on a trained model. Template matching methods, such as [60], match the detected ROI with a previously recorded template in the dataset. Usually some feature points, along the contour of the objects or where the gradient magnitudes are largest, are matched to find the best template. Models based on PPF [11,61] describe the relative position and orientation of two oriented points. This methodology is similar to surflet-pair relations histogram [62]. They calculate the surflet-pairs for the global model and scenes and then retrieve the matched vectors using a hash table. Finally, models based on 3d local features, [63,64], rely on 3d shape descriptors such as local surface orientation and the surface normal vectors. 3d local feature based methods usually work best for LIDAR datasets, but their time complexity can be relatively high. The BOP benchmark suggests that PPF and template based methods outperform the learning based and 3d local features approaches with respect to detection accuracy. However, 3d local features can be quite time consuming compared to the learning-based approaches. Handling the partially or heavily occluded scene is still an open problem. The BOP benchmark did not evaluate the Point Feature Histogram (PFH) method, which was initially introduced in [44,65]. However, this method is very similar to the PPF approach. The 4d feature vectors of PPF and PFH are shown in Figure 4a and Figure 4b, respectively.

**Figure 5:**
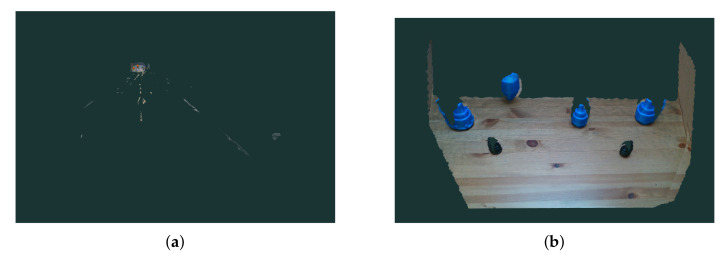
(**a**) Original point cloud captured by a RGB-D sensor, (**b**) the same point cloud after removing the unwanted region.

The main difference between the two is that PPF is a semiglobal approach and the surflet-pair feature vectors are extracted from both the reference model and the captured scene. They use a look-up table to find the closest match between the scene and the model. However, PFH is a local approach based on surflet-pair feature. They store the feature vectors in a 16 bin histogram. In 2018 Buch et al. [66] proposed that histogram based PPF can work relatively well for various textured and heterogeneous datasets. In this work we propose a combined PFH and template matching method to address the 6d pose estimation. To get the 3 rotation and 3 translation values we need to use depth information. Point cloud data is usually noisy and contains unwanted regions and points. Therefore, a preprocessing step is needed to make the data usable. The preprocessing pipeline consists of cropping, background subtraction, filtration and ROI extraction. As Figure 5a shows, the original captured point cloud can contain unwanted areas. We crop the original point cloud using a box filter. The cropped point cloud contains only the ROI. The parameters for the box filter are determined empirically based on the scene setup.

**Figure 6:**
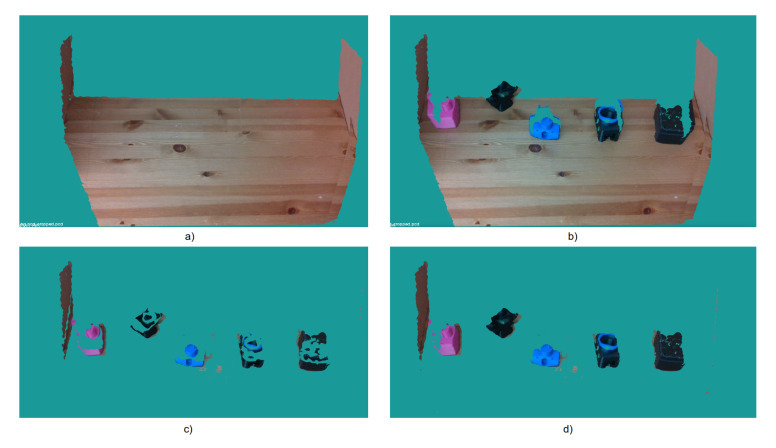
(**a**) Background point cloud, (**b**) Scene point cloud, (**c**) Background subtraction without adding background to scene, (**d**) Background Subtraction after adding background to scene.

In order to obtain an accurate ROI extraction, a point cloud of the background, is captured, cropped and stored. When a new scene is loaded using the current setup, the background point cloud is first added to the captured point cloud (after cropping) and then passed to a Random Sample Consensus (RANSAC) Segmentation model. The model extracts the planes in the point cloud, which are then removed from the combined point cloud. Adding the background to the captured scene ensures that occluded areas of the background not captured are in fact presented during the background subtraction stage. Figure 6 shows the result of background subtraction.

As shown in Figure 6d, the result of background subtraction contains a few patches of unwanted points. To eliminate the unwanted points, a statistical outlier removal algorithm is used. It uses the statistical information of neighbourhood points to remove outlier data. This approach utilises the average distance of each point along with the standard deviation of distance from its k nearest neighbours (50 nearest neighbours are used in our approach) to classify the points as outliers [65]. To extract ROI for objects in the scene, we use the regions provided by the optical approach. To get an accurate correspondence between the pixel location in the optical image and index location in the point cloud data we add an offset, which is the difference in the locations of optical and depth sensors. To find the 6d pose of an already recognised object, we perform 3d template matching between the extracted ROI and templates of that specific class in the database. Our template matching is based on 4d geometric features as first introduced in [62] and Fast Point Feature Histogram (FPFH) [4]. The geometrical features are extracted from neighbourhood surflet pairs, ($p_{i}$,$n_{i}$) and ($p_{j}$,$n_{j}$), where $p_{i}$ and $p_{j}$ are the 3d points at location *i* and *j* of the point cloud, and $n_{i}$ and $n_{j}$ are the corresponding surface normals respectively. The most significant eigenvector of the covariance matrix of neighbouring points around point *p* represents the surface normal at that point. Equation 4 explains the computation.

(4) $$C=\frac{1}{k}\sum_{i=1}^{k}\left(p_{i}-\bar{p}\right)\cdot {\left(p_{i}-\bar{p}\right)}^{T}$$

where *C* is the covariance matrix of *k* nearest neighbour points with a fixed radius *r* around point *p*. It is important to reorient the points properly to handle the flipping problem of close points, as described in [67]. The extracted geometrical features are invariant to translation and rotation. Later [44,65] introduced Point Feature Histogram (PFH) by adding local histogram to 4d geometrical features for more accurate point labelling and feature extraction. Even though PFH features are robust, they can create bottleneck issues due to high computational complexity. As discussed by the authors, the theoretical complexity of PFH is $O(n\cdot k^{2})$, where *n* is the number of points in the point cloud and *k* is the number of neighbours for each point. In [4], the authors introduced FPFH features, which retain most of the power of PFH but the computational complexity is reduced to O(n·k). All the segments of the scene are processed sequentially and are aligned to the preprocessed template files in the database using Sample Consensus Initial Alignment, described in [4]. For each of the templates, we use the algorithm to minimise the distance between the samples and calculate a fitness score and transformation detail. The acceptable translation and rotation error ranges are smaller than 5 cm and 5° respectively as described in [68] and used by [22]. These ranges are justifiable, compared to our capturing scenes which are around 5 $m^{2}$ to 6 $m^{2}$. The rotation matrix and translation vector for the best aligned and second-best aligned templates are then returned.

## 4. Experimental Setup and Data Acquisition

**Figure 7:**
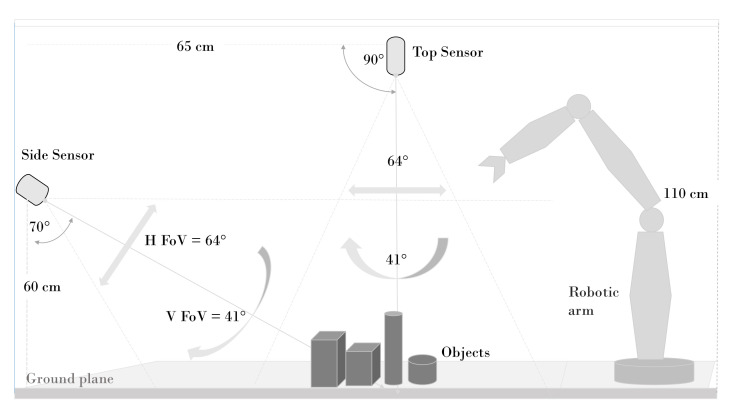
Experimental setup. Top sensor provides an initial estimate of the ROI and the images from the side view camera provide a final object detection and recognition.

**Figure 8:**
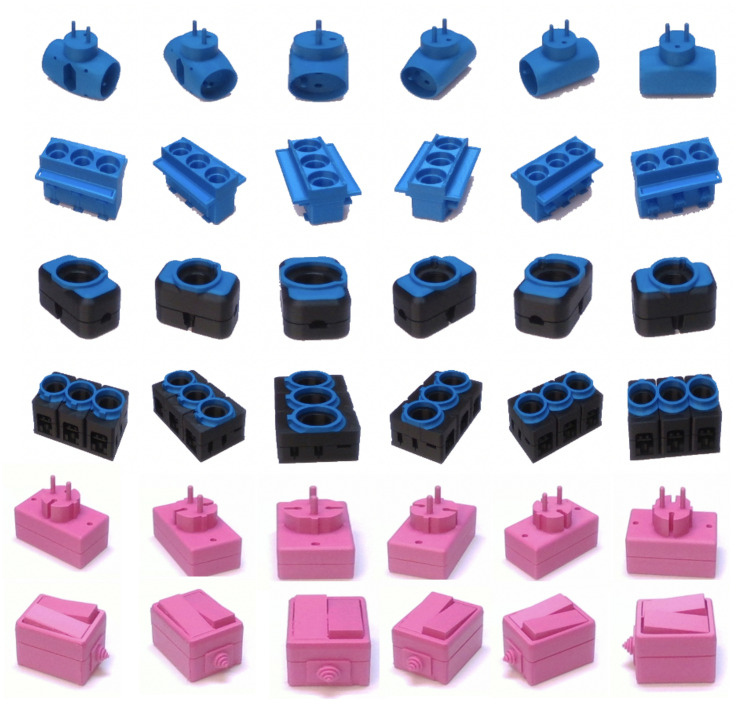
A subset of training set corresponding to objects 5, 7, 9, 21, 22 and 25 of the Tless dataset.

We used two Intel RealSense D415 cameras for image acquisition as shown in Figure 7. Intel RealSense D415 uses an optical image sensor to capture regular RGB images and stereo vision to calculate depth. The camera offers high RGB-D resolution capabilities and is very light and easy to use in industrial environments. To capture the training dataset, we only used one of the cameras and fixed it with a tripod in front of an electric turntable. The camera must be placed with a minimum distance of 20 cm from the scene according to the sensor specifications. We placed an object, from 30 TLess objects, at four different locations of the turntable and captured a sequence of images by rotating the turntable at 1-degree intervals for each location. We also added shear and zoom transformation to the captured images in order to augment the training data. Therefore, our training set consists of 172,800 images. The training images are cropped to the bounding box around each object and each image is normalised and resized to 60×60. Figure 8 shows a subset of the training set for six different objects. We also created a database for template matching and pose estimation, which consists of twelve point clouds, corresponding to a sequence of images taken at 30 degree intervals, for each of the 30 objects.

All the objects are 3d printed using PLA material in different colours. The industrial CAD models for 3d printing were obtained from the TLess dataset [25]. The setup was arranged in an enclosed environment to control the illumination condition. It is important to note that our proposed approach is robust to environmental changes and can work in different indoor settings. We tested our approach in three different setting as discussed in Section 6.

**Figure 9:**
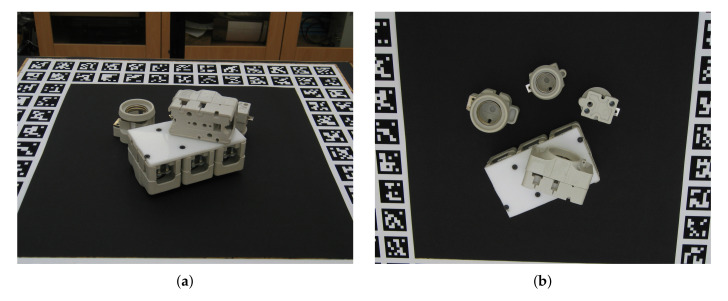
(**a**) Side view perspective and (**b**) top view perspective on TLess scene.

Two sensors were used for testing purpose: one sensor on the side and one on top, as shown in Figure 7. The side sensor is located at a height of 60 cm from the capturing surface and an angle of 70 with respect to the ground surface. The sensor at the top of the scene is located 110 cm from the capturing surface and with the horizontal axis parallel to the ground. On the right side is the robotic arm that performs pick-and-place object tasks. Both sensors have field of view FOV (H × V × D with range of 69.4${}^{{}^{\circ}}$× 42.5${}^{{}^{\circ}}$× 77${}^{{}^{\circ}}$ +/− 3${}^{{}^{\circ}}$) to capture the area where objects are placed. This configuration allows recognition but also handles occlusions between objects by having two completely different perspectives of the same scene. If an object is not located from the side sensor or partially occluded, it can be located by the other sensor. This benefits the utility and efficiency of our system. Figure 9 shows two images from a TLess dataset scene to demonstrate the effectiveness of this configuration. The first image, Figure 9a, is captured from the side, and the second image, Figure 9b, is captured from the top. Note that the objects in Figure 9a are quite occluded. In contrast, Figure 9b shows the same scene from another perspective where all objects are fully visible. This shows the benefit of using multiple cameras.

## 5. Evaluation Metrics

We describe the evaluation metrics used to validate our algorithm in this section. The system’s performance for object detection is measured in terms of intersection over union (IoU). This metric describes the intersection between the predicted bounding box $B_{P}$ of the target object and the ground truth-bounding box $B_{R}$, which is represented by the following equation:

(5) $$IoU=\frac{B_{R}\cap B_{P}}{B_{R}\cup B_{P}}$$

Objects are considered properly detected in the image if IoU > 0.7. For the 6d pose, we evaluate the sum of squared distances (SSD) between the predicted pose point cloud and the ground truth point cloud of the object in the scene.

## 6. Experimental Results and Comparison

### 6.1. Experimental Results on Object Recognition

**Figure 10:**
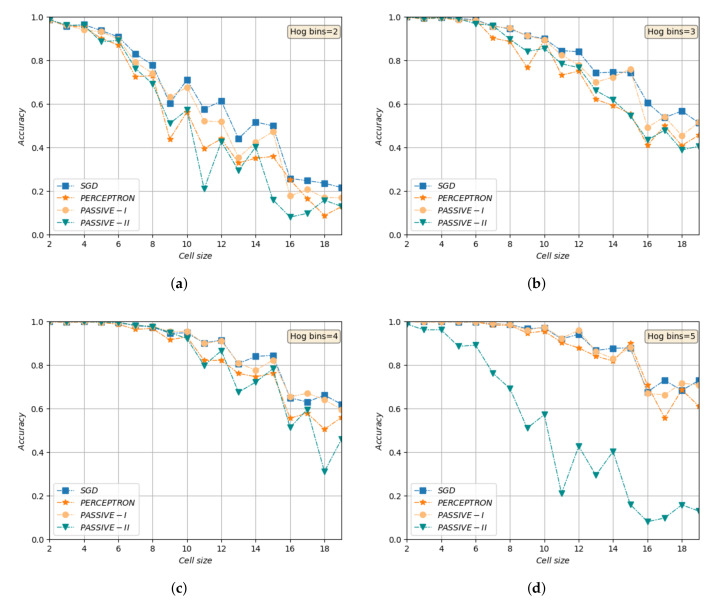
Object recognition accuracy of different classifiers vs. different HOG cell size, range [2,20], for (**a**) 2 bins histofram, (**b**) 3 bins histogram, (**c**) 4 bins histogram (**d**) 5 bins histogram.

We run various experiments, to find the best parameters and classifier considering both validation accuracy and time efficiency. Figure 10 shows accuracy vs. HOG cell size for different bin sizes. As it was expected and obvious from these plots, a smaller cell and more histogram bins will result in a denser feature space and higher accuracy. However, the training time will increase significantly too. We chose cell size 6×6 and histograms with 4 bins based on our experiments as the best trade-off. As Figure 10c shows, different models perform well with these parameters and the training times are satisfactory.

**Figure 11:**
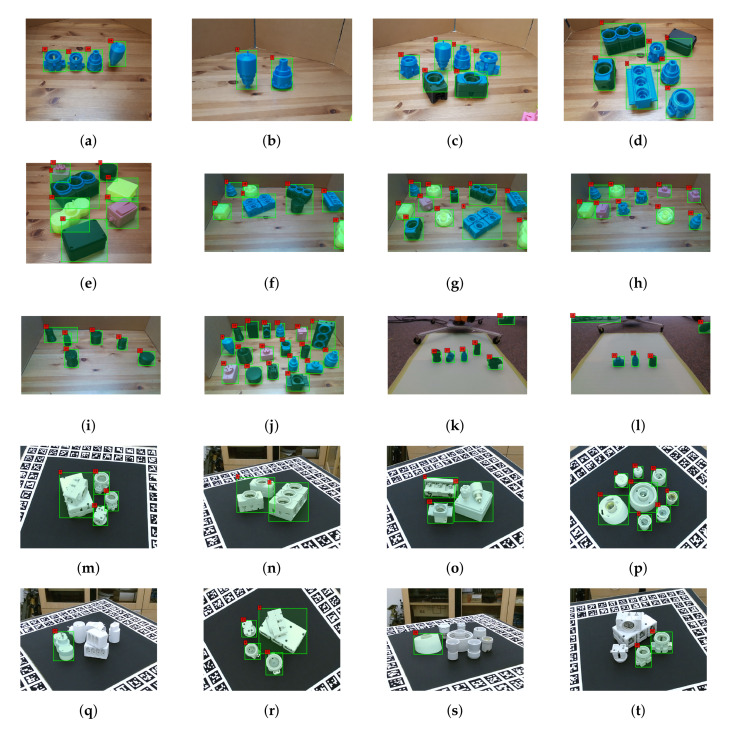
(**a**–**j**) Recognition results on our first setting’s test scenes, (**k**,**l**) recognition results on our second setting’s test scenes, and (**m**–**t**) recognition results on Tless test scenes.

We have tested our algorithm on 30 different test scenes, from two different indoor settings with different backgrounds and illumination conditions. Table 1 reports the accuracy for individual objects with an average accuracy of 70.43% for our own test scenes. To provide a better picture to the readers, we also tested our model on the Primescene test scenes of the Tless dataset using scenes with similar camera angles as our training set. We present the recall scores of our results and those from [12] on Tless in Table 1. However, it should be noted that in [12], they trained their model on the full training set of Tless which covers 270 degrees orientation in 3 axes. Their reported recall scores correspond to the model trained with the RGB images of reconstructed objects and the average accuracy is 19.26%. On the other hand, we do not need the arbitrary orientation as we focus on pick and place tasks in robotics environments and our training set only covers rotation around the vertical axis. Therefore, we tested our method on the images with similar camera angles as our experimental setup. The reason for lower accuracy in Tless test scenes is due to the arbitrary orientations of the objects and complete or heavy occlusion on some of the images. Some recognition results of our test scenes and Tless test scenes are shown in Figure 11a–f and Figure 11m–t respectively. The results also show that our model can handle occlusion reasonably. However, in a heavily occluded scene, such as Figure 11f, object segmentation fails to extract the ROI accurately.

### 6.2. Experimental Results on Pose Estimation

**Figure 12:**
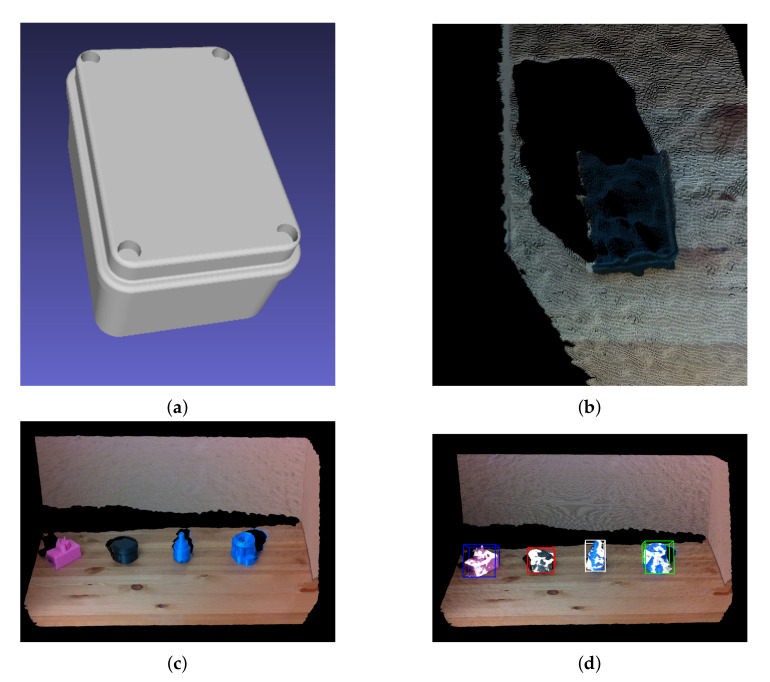
(**a**) CAD model for Object 29, (**b**) an incomplete point cloud representation, (**c**) a scene with 4 objects from the dataset and (**d**) localisation of point clouds as shown by the aligned bounding boxes.

Our pose estimation algorithm is invariant to lighting conditions. This results in high accuracy if the point cloud is captured precisely. In some scenarios, due to reflection, the depth sensor is not able to register the surface of an object, which results in an incomplete point cloud as shown in Figure 12b. Therefore, we need to capture dense point cloud with high precision in order to address this problem. Figure 12c,d show one such captured scene along with the detected pose and aligned point cloud.

**Figure 13:**
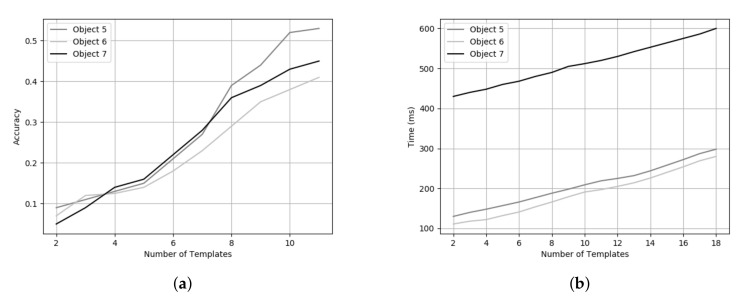
(**a**) Pose estimation accuracy vs. number of templates used for Objects 5, 6 and 7 in Scene 2. (**b**) Runtime vs. number of templates used for Objects 5, 6 and 7 in Scene 2.

**Figure 14:**
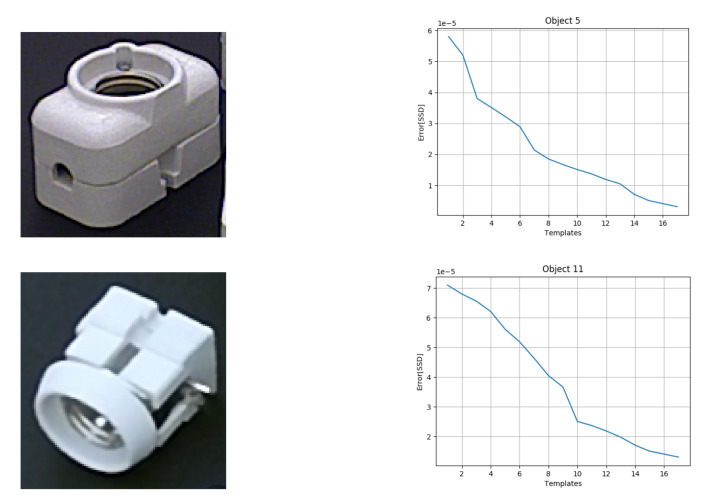
Comparing error function (SSD) vs. number of templates on Object 5 (**Top row**) and Object 11 (**bottom row**).

**Figure 15:**
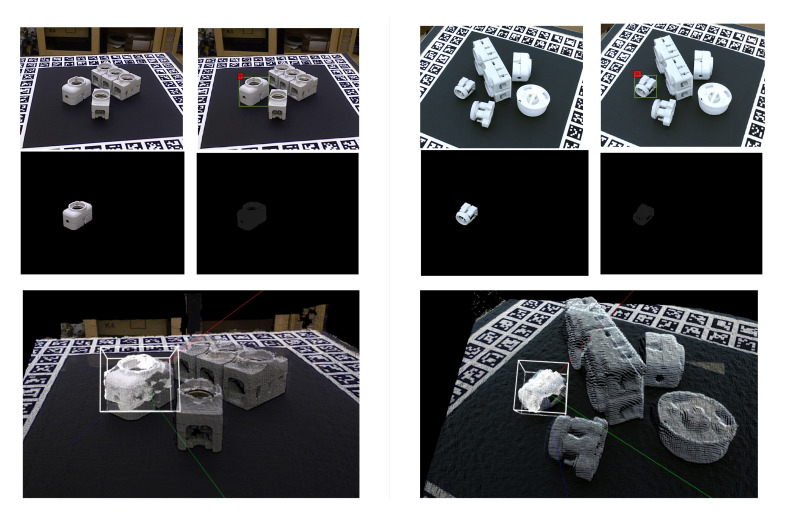
**Left**: Top row shows RGB input image and the predicted bounding box for object 5 Scene 2, middle row shows segmented region on RGB and Depth image, and bottom row shows the final predicted pose in the point cloud scene of the input RGB-D image. **Right**: Top row shows RGB input image and the predicted bounding box for object 11 Scene 6, middle row shows segmented region on RGB and Depth image, and bottom row shows the final predicted pose in the point cloud scene of the input RGB-D image.

For each object, we have 12 templates in the dataset captured using a rotating table at 30-degree intervals. The reason behind this rotation interval is based on the trade off between accuracy (Figure 13a) and processing time (Figure 13b). Even though a high number of templates for each object results in a better pose estimation, the time taken increases significantly. To the best of our knowledge, this is the first approach presenting results on the TLess dataset using point clouds to predict the object pose. However, we can also demonstrate the efficiency of our method on different TLess sequences. We tested our pipeline on Scene 2 (Object 5) and Scene 6 (Object 11) of the TLess dataset. The first set of experiments focused on the influence of the number of templates in the database to find the optimal pose for the object in a specific image. We performed the comparison using an increasing number of templates in the range of 2 to 18 to observe the influence on the error sum of squared distances between the predicted point cloud and the reference point cloud, as shown in Figure 14. We found that a high number of templates results in a better pose estimation of the object. We also test our method on all the frames where the object is visible and compare our method with the ground truth bounding box (BBOX). Comparison results are shown in Table 2. Examples of the final pose for Object 5 and Object 6 are shown in Figure 15.

### 6.3. Runtime Analysis

Our current RGB-D implementation is on a platform using Intel Core i7-4790 CPU 3.60 GHz × 8 thread of execution. It takes approximately 215 ms to estimate the final 6d pose of one object. Computational results are shown in Table 3 and Table 4. The computational time can vary due to factors such as multiple objects in the scene, different object sizes and number of templates used. However, it is possible to optimise the different stages in our future work.

## 7. Conclusions

In this paper we proposed a framework to accurately detect and recognise textureless objects, which are common in the manufacturing industry. Recognition was based on estimating 6d pose of an object using RGB-D data. This approach is quite effective for industrial assembly lines and pick-and-place tasks, where a robotic arm is often used to pick a similar, textureless, homogeneous object and place it in its designated location. Textureless manufacture parts make the object recognition tasks challenging. We showed that our proposed method is quite accurate and robust to illumination and background changes. Our object recognition and pose estimation technique is fast and suitable for real-time applications. However, our point cloud pose estimation needs to be improved for real-time and interactive applications. In future work, we will estimate the pose from optical and depth information directly to improve the time performance.

## Figures and Tables

**Table 1:** Object recall for $err_{vsd}<0.3$ on our 30 different test scenes and Primescene test scenes of Tless dataset. Some of our test scenes are shown in Figure 11a–l.

(Obj.#)	Our Method on Our Test Scenes	Our Method Tless Test Scenes	Augmented Autoencoder [12]
1	62.50%	4.16%	9.48%
2	66.67%	5.55%	13.24%
3	42.86%	4.86%	12.78%
4	80.00%	3.47%	6.66%
5	58.33%	16.20%	36.19%
6	55.56%	13.88%	20.64%
7	71.43%	19.44%	17.41%
8	75.00%	15.27%	21.72%
9	62.50%	12.50%	39.98%
10	66.67%	72.22%	13.37%
11	77.78%	11.12%	7.78%
12	87.50%	9.72%	9.54%
13	76.92%	6.94%	4.56%
14	75.00%	5.55%	5.36%
15	71.43%	9.72%	27.11%
16	46.15%	8.33%	22.04%
17	66.67%	5.55%	66.33%
18	83.33%	3.70%	14.91%
19	75.00%	2.77%	23.03%
20	62.50%	4.16%	5.35%
21	71.43%	5.55%	19.82%
22	87.50%	9.72%	20.25%
23	71.43%	48.61%	19.15%
24	83.33%	15.97%	4.54%
25	85.71%	2.77%	19.07%
26	70.00%	4.16%	12.92%
27	75.00%	8.33%	22.37%
28	71.43%	5.55%	24.00%
29	66.67%	11.11%	27.66%
30	66.67%	18.05%	30.53%

**Table 2:** Pose Estimation Results on TLess Dataset- (Object 5 and Object 11).

Experiment	TLess Dataset	
	Object 5	Object 11
Crivellaro [21] + GT BBOX	0.19	0.21
Vidal et al. [61]	0.69	0.69
Sundermeyer et al. [12] no color augmentation	0.47	–
GT BBOX + our pose method (12 templates)	0.68	0.58
Our method (2 templates)	0.08	0.05
Our method (4 templates)	0.14	0.012
Our method (8 templates)	0.23	0.24
Our method (10 templates)	0.39	0.37
Our method (12 templates)	0.53	0.45

**Table 3:** Performance time of the different processes in our pipeline.

	CPU
Watershed + HED + CIE-LAB	10 ms
HOG + SGD	15 ms
HOG + PERCEPTRON	15 ms
HOG + PASSIVE AGRESSIVE I	15 ms
HOG + PASSIVE AGRESSIVE II	15 ms
FPFH	190 ms

**Table 4:** Performance time for single object pose estimation.

Method	fps
Vidal et al. [61]	0.2
Brachmann et al. [24]	2
Kehl et al. [58]	2
BB8 [22]	4
Our proposed method	5
Crivellaro et al. [21]	10
SSD6D [23]	12
Sundermeyer et al. [12] (SSD)	42
[12] (RetinaNet)	13
Tekin et al. [69]	50
